# Cobalt Chloride Enhances the Anti-Inflammatory Potency of Human Umbilical Cord Blood-Derived Mesenchymal Stem Cells through the ERK-HIF-1*α*-MicroRNA-146a-Mediated Signaling Pathway

**DOI:** 10.1155/2018/4978763

**Published:** 2018-09-05

**Authors:** Jihye Kwak, Soo Jin Choi, Wonil Oh, Yoon Sun Yang, Hong Bae Jeon, Eun Su Jeon

**Affiliations:** Biomedical Research Institute, R&D Center, and MEDIPOST Co., Ltd., 21 Daewangpangyo-ro 644 Beon-gil, Bundang-gu, Seongnam-si, 13494 Gyeonggi-do, Republic of Korea

## Abstract

Human mesenchymal stem cells (hMSCs), including human umbilical cord blood-derived mesenchymal stem cells (hUCB-MSCs), which have high proliferation capacity and immunomodulatory properties, are considered to be a good candidate for cell-based therapies. hMSCs show enhanced therapeutic effects via paracrine secretion or cell-to-cell contact that modulates inflammatory or immune reactions. Here, treatment with cobalt chloride (CoCl_2_) was more effective than naïve hUCB-MSCs in suppressing inflammatory responses in a coculture system with phytohemagglutinin- (PHA-) activated human peripheral blood mononuclear cells (hPBMCs). Furthermore, the effect of CoCl_2_ is exerted by promoting the expression of anti-inflammatory mediators (e.g., PGE_2_) and inhibiting that of inflammatory cytokines (e.g., TNF-*α* and IFN-*γ*). Treatment of hUCB-MSCs with CoCl_2_ leads to increased expression of microRNA- (miR-) 146a, which was reported to modulate anti-inflammatory responses. Hypoxia-inducible factor- (HIF-) 1*α* silencing and ERK inhibition abolished CoCl_2_-induced miR-146a expression, suggesting that ERK and HIF-1*α* signals are required for CoCl_2_-induced miR-146a expression in hUCB-MSCs. These data suggest that treatment with CoCl_2_ enhances the immunosuppressive capacity of hUCB-MSCs through the ERK-HIF-1*α*-miR-146a-mediated signaling pathway. Furthermore, pretreatment of transplanted MSCs with CoCl_2_ can suppress lung inflammation more than naïve MSCs can in a mouse model of asthma. These findings suggest that CoCl_2_ may improve the therapeutic effects of hUCB-MSCs for the treatment of inflammatory diseases.

## 1. Introduction

Human mesenchymal stem cells (hMSCs), also termed as stromal cells, are isolated from a variety of tissues, including bone marrow, adipose tissue, and umbilical cord blood and are recognized as a promising therapeutic agent for clinical application because of their high proliferative capacity, multilineage differentiation potential, and immunomodulatory properties [1–3]. The release of paracrine/autocrine factors is a key mechanism of action of hMSCs [4–6]. Many studies have demonstrated that transplanted MSCs help prepare the inflammatory microenvironment by producing immunomodulatory factors that modulate the progression of inflammation [1, 2, 7]. Improvement of immunomodulating properties is expected to enhance the therapeutic effects of hMSCs. Hence, the aim of the present study was to develop a method to enhance the immunosuppression of hMSCs and clarify the therapeutic effects of modified hMSCs.

Hypoxia plays pivotal roles in the maintenance of hMSCs and is regulated by several transcriptional factors, including various hypoxia-inducible factors (HIFs) [8]. Of these, HIF-1*α* plays a key role in the cellular response against hypoxia by activating the transcription of various genes involved in the differentiation, colony formation, proliferation, and paracrine action of hMSCs [9]. Cobalt chloride (CoCl_2_) is a hypoxia-mimetic compound that induces biochemical and molecular responses similar to those observed under hypoxic conditions [10]. Treatment with the hypoxia-mimetic CoCl_2_ is used to evaluate the effect of immune responses and delineate the underlying signaling mechanisms [11]. Also, CoCl_2_ has been shown to confer a protective effect on TNF-*α*/IFN-*γ*-induced inflammation *in vitro* [12].

In the present study, the role of CoCl_2_ in the immunomodulation of human umbilical cord blood-derived mesenchymal stem cells (hUCB-MSCs) was examined. Treatment with CoCl_2_ was found to increase the anti-inflammatory effects of hUCB-MSCs in a HIF-1*α*- and ERK-dependent manner. Furthermore, CoCl_2_-induced microRNA- (miR-) 146a expression regulated the secretion of inflammatory cytokines, whereas anti-miR-146a abolished CoCl_2_-induced anti-inflammatory properties of hUCB-MSCs. These results demonstrate for the first time that miR-146a is critical for the CoCl_2_-induced anti-inflammatory properties of hUCB-MSCs.

Studies of rodent asthma models demonstrated that intravenous administration of MSCs attenuated the major pathologic features of asthma, including airway inflammation [13, 14] and remodeling, and the enhanced anti-inflammatory capacity of MSCs increased the therapeutic effect in an *in vivo* asthma model [15, 16]. Moreover, CoCl_2_ preconditioning was shown to improve the therapeutic effects of hUCB-MSC in asthma. These results suggest that CoCl_2_ signaling may improve the therapeutic effects of hUCB-MSCs.

## 2. Materials and Methods

### 2.1. Materials

Alpha-minimum essential medium (MEM) and fetal bovine serum (FBS) were purchased from Gibco (Carlsbad, CA, USA). Trypsin, phosphate-buffered saline (PBS), and distilled water were purchased from Biowest (Carlsbad, CA, USA). Lipofectamine™ 3000 reagent was purchased from Invitrogen Corporation (Carlsbad, CA, USA). Antibodies against phospho-ERK and ERK were obtained from Cell Signaling Technology (Beverly, MA, USA), those against HIF-1*α* were purchased from BD Biosciences (Oxford, UK), and those against glyceraldehyde 3-phosphate dehydrogenase (GAPDH) were obtained from Gwangju Institute of Science and Technology (Gwangju, Korea). CoCl_2_ was purchased from Sigma-Aldrich Corporation (St. Louis, MO, USA). U0126 and peroxidase-labeled secondary antibodies were purchased from Cell Signaling Technology (Beverly, MA, USA).

### 2.2. Cell Culture and Treatment

hUCB-MSCs were collected from the umbilical cord vein of a newborn baby, with the consent of the mother. To isolate and expand MSCs from cord blood, mononuclear cells were removed using Ficoll–Hypaque solution (*d* = 1.077 g/cm^3^; Sigma-Aldrich Corporation) and MSCs were then seeded at 5 × 10^5^ cells/cm^2^ in culture flasks. After the formation of colonies, spindle-shaped cells were reseeded for expansion. hUCB-MSCs were cultured in alpha-MEM supplemented with 10% FBS and gentamicin in a humidified 5% CO_2_ atmosphere at 37°C. Cells were passaged to 80%–90% confluency and either used for experiments or redistributed to new culture plates. All experiments were performed with cells that were passaged 5–8 times.

To prepare CoCl_2_ stock solution, the chemical was dissolved directly in distilled water (100 mM). The stock solutions were filter-sterilized (0.22 mm) and stored at −20°C. Cells were cultured in alpha-MEM supplemented with 10% FBS and gentamicin at 37°C under humidified 5% CO_2_ atmosphere. CoCl_2_, a chemical hypoxia-mimetic agent, was added into the medium at 100 *μ*M, and cells were incubated in the presence of CoCl_2_ for the indicated times and then used for further assays. In order to clarify the role of ERK1/2 in CoCl_2_-enhanced immunosuppressive capacity of hUCB-MSCs, cells were pretreated with U0126 (ERK1/2 inhibitor) for 60 min prior to treatment with 100 *μ*M CoCl_2_. The effects of HIF-1*α* knockdown were observed by treatment with 100 *μ*M CoCl_2_ at 48 hr posttransfection with HIF-1*α*-specific siRNA.

### 2.3. Mixed Lymphocyte Reaction (MLR) and Enzyme-Linked Immunosorbent Assay (ELISA)

Prior to the MLR process, stimulator hPBMCs and hUCB-MSCs were inactivated by treatment with 10 *μ*g/mL mitomycin C (Sigma, St. Louis, MO) for 1 hr at 37°C. hUCB-MSCs (1 × 10^3^ cells/well) were seeded and maintained at 37°C in a humidified incubator for 2–4 hr and then cocultured with responder peripheral blood mononuclear cells (PBMCs; 1 × 10^5^ cells/well; AllCells, Boston, MA, USA) and stimulator PBMCs (1 × 10^5^ cells/well; AllCells) from different donors. Phytohemagglutinin- (PHA-) (5 *μ*g/ml, Roche) treated PBMCs were used as the positive control. After coculturing with MSCs, PBMCs were maintained for 6 days in Roswell Park Memorial Institute 1640 medium (Gibco) supplemented with 10% FBS and gentamicin. Proliferation of PBMCs was measured using a cell proliferation BrdU (colorimetric) ELISA kit (Roche). Supernatant was collected after the MLR assay to measure levels of the immunoregulatory cytokines TNF-*α*, IFN-*γ*, and PGE_2_ using ELISA kits (R&D Systems Inc., Minneapolis, MN, USA).

### 2.4. Cell Proliferation Assay

The MTS ([3-(4,5-dimethylthiazol-2-yl)-5-(3-carboxymethoxyphenyl)-2-(4-sulfophenyl)-2H-tetrazolium, inner salt]) cell proliferation assay was used to detect the effect of drug sensitivity. Briefly, hUCB-MSCs were seeded in 96-well plates and incubated at 37°C in a 5% CO_2_ incubator for 24 hr. The cells were treated with various concentrations of CoCl_2_ for 72 hr. Then, the MTS assay was performed using CellTiter 96 AQueous One Solution (Promega Corporation, Madison, WI, USA) following the manufacturer's instructions.

### 2.5. Western Blot Analysis

After treatment as described above, hUCB-MSCs were washed with ice-cold 1x PBS and lysed with radioimmunoprecipitation assay buffer containing protease inhibitors and phosphatase inhibitors (Roche, Basel, Switzerland). Protein concentrations were determined with the Bradford assay. Lysates were separated using Novex*®*, NuPAGE*®*, and Bolt® precast gels (Invitrogen Corporation) under denaturing conditions and transferred to nitrocellulose membranes. After blocking with 1% bovine serum albumin solution, membranes were immunoblotted with various antibodies and then probed with horseradish peroxidase-conjugated secondary antibodies. Bands were visualized with an enhanced chemiluminescence immunoblotting system (GE Healthcare Life Sciences, Chicago, IL, USA).

### 2.6. Transfection with Small Interference RNA (siRNA) or miR and Quantitative Real-Time PCR (RT-qPCR) Analysis

The following oligoribonucleotides for RNA interference were synthesized, desalted, and purified by ST Pharm. Co. Ltd. (Siheung, Gyeonggi, Korea): HIF-1*α* siRNA S5′-GUC CCA UGA AAA GAC UUA AdTdT-3′ and A5′-UUA AGU CUU UUC AUG GGA CdTdT-3′ and nonspecific control siRNA S5′-GGA GAA AUG GUG CGA GAA GdTdT-3′ and A5′-CUU CUC GCA CCA UUU CUC CdTdT-3′. For siRNA experiments, hUCB-MSCs were transfected with 100 nM HIF-1*α* or control siRNAs with Lipofectamine 3000 reagent (Invitrogen Corporation) according to the manufacturer's instructions. Cells were cultured in growth medium for 48 h, and the knockdown efficiency of target genes was confirmed by determining the decrease in the expression level of total HIF-1*α*.

Precursor miR-146a (pre-miR-146a) and antisense miR-146a (anti-miR-146a) were purchased from Ambion Inc. (Austin, TX, USA) and used for activation or inhibition of miR function, respectively. To determine the expression levels, miR was isolated using TRIzol reagent (Invitrogen Corporation) according to the manufacturer's protocol. The level of miR-146a was determined using stem loop-specific RT primer and TaqMan PCR Master Mix (Applied Biosystems, Carlsbad, CA, USA) and normalized against the level of U6 snRNA.

### 2.7. Flow Cytometry

For flow cytometry, single-cell suspensions were generated from 1 × 10^6^ cells that were incubated with the indicated monoclonal antibody (mAb) at room temperature for 15 min. The following conjugated antibodies were used for the analyses: fluorescein isothiocyanate-conjugated mAbs against CD14, CD45, CD34, and HLA-DR and phycoerythrin-conjugated mAbs against CD90, CD105, CD73, and CD166 (BD Biosciences). After incubation for 15 min, cells were washed twice with Dulbecco's PBS and fixed with 1% paraformaldehyde. At least 10,000 events were measured using a fluorescence-activated cell sorting (FACS) instrument (FACSCalibur; Becton Dickinson, San Jose, CA, USA), and cell flow cytometry data were analyzed using CellQuest software (Becton Dickinson). A fluorescence histogram for each MSC marker was marked with the control antibody. The percentages of positive cells were subtracted from the isotype control antibody of each conjugate.

### 2.8. Generation of Asthma Model and Evaluation of Lung Inflammation

BALB/c female mice (6 weeks old) were purchased from Orient Bio Inc. (Seongnam, Korea) and acclimated for 1 week prior to beginning the experiment. To induce asthma, mice were anesthetized and then sensitized with 75 *μ*g of ovalbumin (OVA; Sigma-Aldrich Corporation) and 10 *μ*g of polyinosinic-polycytidylic acid [poly(I:C); Calbiochem-Merck KGaA, Darmstadt, Germany] via intranasal administration on days 0, 1, 2, 3, and 7; they were then intranasally challenged with 50 *μ*g of OVA with 10 *μ*g of poly(I:C) on days 14, 21, 22, and 23. To verify the treatment effect of hUCB-MSCs or CoCl_2_-MSCs, mice were intravenously injected into the tail vein on day 15 with hUCB-MSCs or CoCl_2_-MSCs (1 × 10^5^ cells/100 *μ*L/mouse). As the positive control group, several mice were administered equal volumes of PBS. All mice were sacrificed on day 24, and bronchoalveolar lavage fluid (BALF) was obtained from the left lung by lavaging three times with 1 mL of saline via trachea cannula, while the right lung was resected. Then, BALF was centrifuged, precipitated cells were resuspended in 1 mL of PBS, and the number of cells was counted under a biological microscope (Olympus Corporation, Tokyo, Japan). Isolated lungs were fixed with 4% paraformaldehyde, embedded in paraffin, and then cut into sections at a thickness of 3–4 *μ*m, which were stained with hematoxylin and eosin.

### 2.9. Statistical Analysis

All statistical analyses were performed using SPSS software version 18 (SPSS Inc., Chicago, IL, USA), and data are reported as the mean ± standard deviation (SD). Differences and significance were verified by one-way analysis of variance followed by Fisher's least significant difference post hoc test. A probability (*p*) value of <0.05 was considered statistically significant.

## 3. Results

### 3.1. Effects of CoCl_2_ on the Anti-Inflammatory Effects of hUCB-MSCs

**Figure 1:**
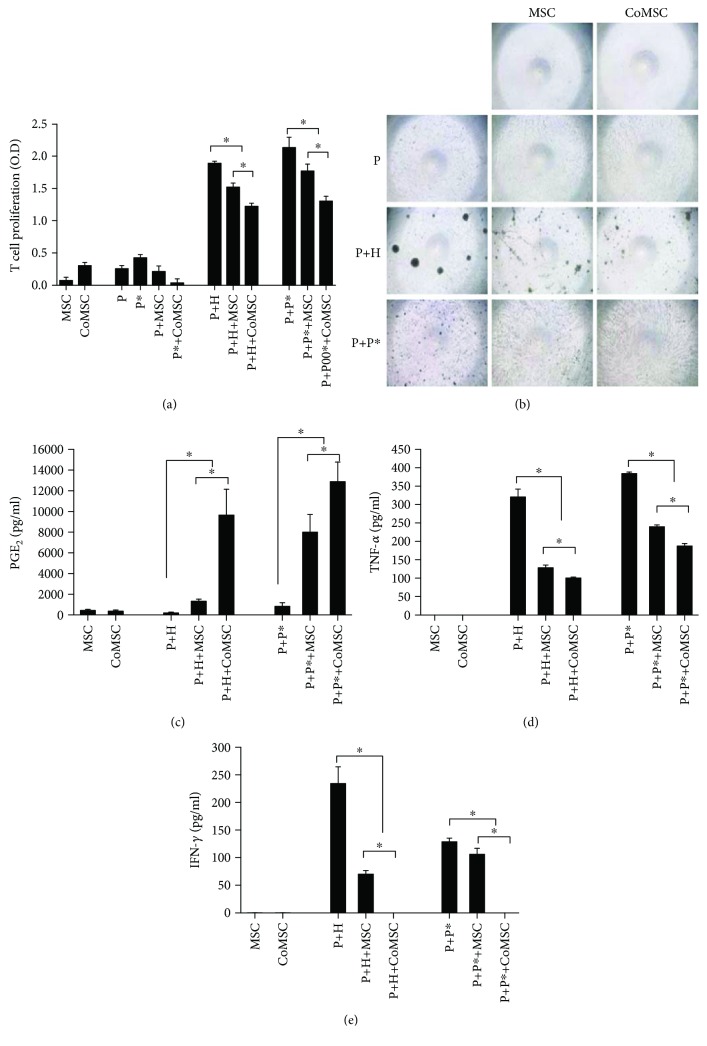
Cobalt chloride (CoCl_2_) stimulates the immunomodulation of human umbilical cord blood-derived mesenchymal stem cells (hUCB-MSCs). (a and b) The immunogenicity of hUCB-MSCs was assessed using the MLR assay. Allogeneic hPBMCs were cocultured with hUCB-MSCs. The proliferation of responding cells was assessed using the MLR assay. PGE_2_ (c), TNF-*α* (d), and IFN-*γ* (e) levels in the MLR culture supernatants were measured by ELISA. Data represent the mean ± SD, *n* = 3; ^∗^ *p* < 0.05. P: hPBMCs; P^∗^: allogeneic hPBMCs as stimulator; H: phytohemagglutinin; MSC: naïve hUCB-MSCs; CoMSC: CoCl_2_-pretreated hUCB-MSCs.

To investigate the effects of CoCl_2_ on the immunomodulatory properties of hUCB-MSCs, MLR was performed. CoCl_2_-treated hUCB-MSCs were prepared as described in Materials and Methods. When CoCl_2_-treated hUCB-MSCs were cocultured with allogeneic hPBMCs or PHA, the proliferation and cluster formation of T cells decreased compared with that of naïve hUCB-MSCs (Figures 1(a) and 1(b), and Supplementary Figure 1). To confirm the immunomodulatory effect, the supernatant from the MLR assay was obtained, and the production of PGE_2_, TNF-*α*, and IFN-*γ* was confirmed with ELISA. The results showed that CoCl_2_-treated hUCB-MSCs highly expressed the anti-inflammatory mediator PGE_2_ (Figure 1(c)), whereas expression levels of the proinflammatory cytokines TNF-*α* and IFN-*γ* (Figures 1(d) and 1(e)) were relatively lower than those in the control group.

### 3.2. The Effects of CoCl_2_ Treatment on Characterization of hUCB-MSCs

**Figure 2:**
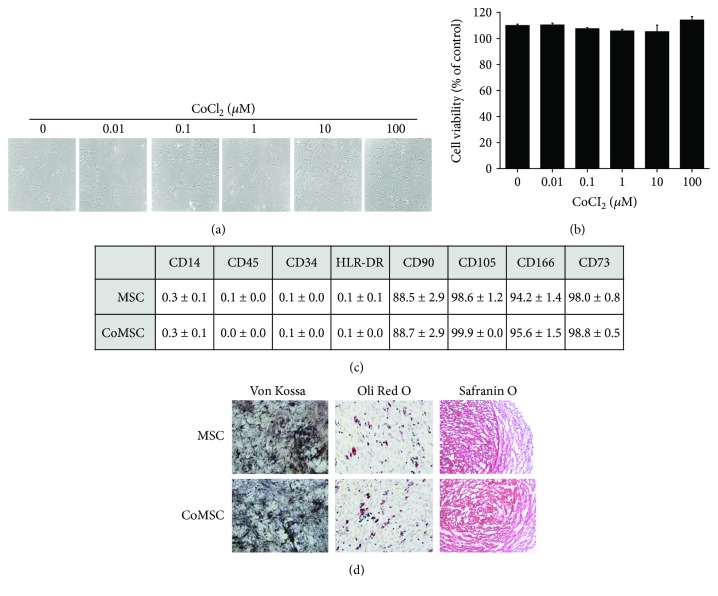
Effects of cobalt chloride (CoCl_2_) on the characterization of hUCB-MSCs. (a and b) The morphology and growth curve of cultured hUCB-MSCs were analyzed. hUCB-MSCs were treated with the indicated concentrations of CoCl_2_ for 72 hr. Phase-contrast image of hUCB-MSCs were obtained with an inverted microscope equipped with a digital camera. Cell viability was determined with the MTS assay. (c) The immunophenotypic characteristics of naïve hUCB-MSCs and CoCl_2_-treated hUCB-MSCs were examined by flow cytometry. (d) During incubation in specialized induction media, multilineage differentiation was measured by staining for typical lineage markers. Osteogenic, adipogenic, and chondrogenic lineages were measured by staining for von Kossa, Oil Red O, or safranin O, respectively. Data represent the mean ± SD, *n* = 3. MSC: naïve hUCB-MSCs; CoMSC: CoCl_2_-pretreated hUCB-MSCs.

To explore whether CoCl_2_ can induce morphological and cell viability changes, hUCB-MSCs were treated with CoCl_2_. As shown in Figures 2(a) and 2(b), treatment with CoCl_2_ had no effect on the morphology or viability of hUCB-MSCs. FACS analysis showed that CoCl_2_-treated hUCB-MSCs expressed the MSC-specific markers CD90, CD105, CD166, and CD73, but not CD14, CD45, CD34, and HLA-DR (Figure 2(c) and Supplementary Figure 2). Next, the effects of CoCl_2_ treatment on the multilineage differentiation of hUCB-MSCs were investigated. MSCs can differentiate into osteoblast, adipocytes, and chondrocytes when cultured in defined media specific for induction of the respective cell types. Osteogenesis was associated with the presence of calcium deposits as shown by von Kossa staining. Adipogenesis was observed by staining of cytoplasmic lipid vacuoles with Oil Red O. Chondrogenesis was observed by an increase in proteoglycans and was demonstrated by safranin O staining (Figure 2(d)). Pretreatment with CoCl_2_ can successfully differentiate hUCB-MSCs into multiple cell types, including osteoblasts and adipocytes, and chondrocytes. These results suggest that CoCl_2_ treatment had no influence on the characterization of hUCB-MSCs.

### 3.3. miR-146a Controls the CoCl_2_-Induced Anti-Inflammatory Effects of hUCB-MSCs

**Figure 3:**
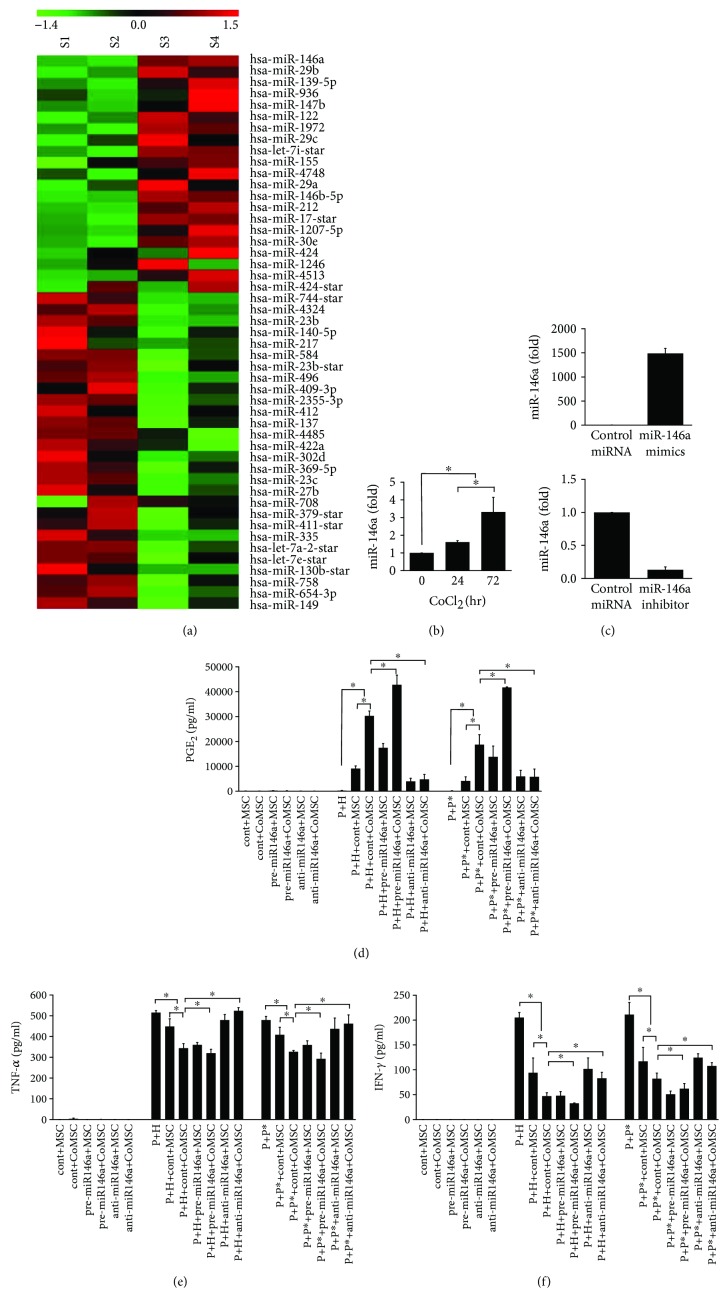
Cobalt chloride- (CoCl_2_-) induced expression of miR-146a in immunomodulation of hUCB-MSCs. (a) hUCB-MSCs (S1–2) and coculture of hPBMCs with hUCB-MSCs (hPBMC + hMSC, S3–4) were collected for microRNA (miR) microarray analysis. Normalized log2 miRNA expression for miRNAs that showed significant differences (*p* < 0.05) between groups is listed according to the color scale. (b) hUCB-MSCs were treated with 100 *μ*M CoCl_2_ and then harvested at the indicated times. The expression level of miR-146a was determined by RT-qPCR. (c) hUCB-MSCs were transfected with scrambled miR-control, miR-146a mimics, or inhibitors. The expression level of miR-146a was determined by RT-qPCR. (d–f) hUCB-MSCs were transfected with scrambled miR-control, miR-146a mimics, or inhibitors and exposed to 100 *μ*M CoCl_2_ for 72 h, and then allogeneic hPBMCs were cocultured with hUCB-MSCs. PGE_2_, TNF-*α*, and IFN-*γ* levels in the culture supernatants were measured by ELISA. Data represent the mean ± SD, *n* = 3; ^∗^ *p* < 0.05. P: hPBMCs; P^∗^: allogeneic hPBMCs as stimulator; H: phytohemagglutinin; MSC: naïve hUCB-MSCs; CoMSC: CoCl_2_-pretreated hUCB-MSCs.

Regulation of inflammatory responses in a disease state is mediated by coordinated control of gene expression via modulation by miRs. To identify potential miR targets, an miR expression profiling experiment was conducted using two cell populations: hMSCs and coculture of hPBMCs with hMSCs (hPBMC + hMSC). miR microarray analysis of these two cell populations was performed using Affymetrix GeneChip miR microarrays (Affymetrix, Santa Clara, CA, USA). The results showed that 21 miRs were significantly upregulated and 28 were significantly downregulated in hPBMC + hMSC compared with those in hMSCs. In particular, miR-146a was upregulated by approximately 53-fold in hPBMC + hMSC (Figure 3(a), Tables 1 and 2). To determine if miRs are involved in CoCl_2_-induced anti-inflammation, hUCB-MSCs were treated with CoCl_2_ and the expression of miR-146a was determined by RT-qPCR. As shown in Figure 3(b), miR-146a was significantly upregulated in CoCl_2_-treated hUCB-MSCs. To determine whether miR-146a expression is critical for CoCl_2_-induced anti-inflammation, hUCB-MSCs were transfected with control siRNA, pre-miR-146a, or anti-miR-146a (Figure 3(c)), followed by treatment with CoCl_2_ for 72 hr. As previously shown, expression of an anti-inflammatory mediator, such as PGE_2_, increased, whereas that of proinflammatory factors TNF-*α* and IFN-*α* decreased in CoCl_2_-treated hUCB-MSCs. However, as shown in Figures 3(d)–3(f), CoCl_2_-regulated expression levels of these factors significantly changed in anti-miR-146a-transfected hUCB-MSCs. Furthermore, hUCB-MSCs transfected with pre-miR-146a showed increased expression of PGE_2_ and decreased expression of TNF-*α* and IFN-*γ*.

### 3.4. CoCl_2_ Increased the Expression of miR-146a through ERK-HIF-1*α*-Dependent Signals

**Figure 4:**
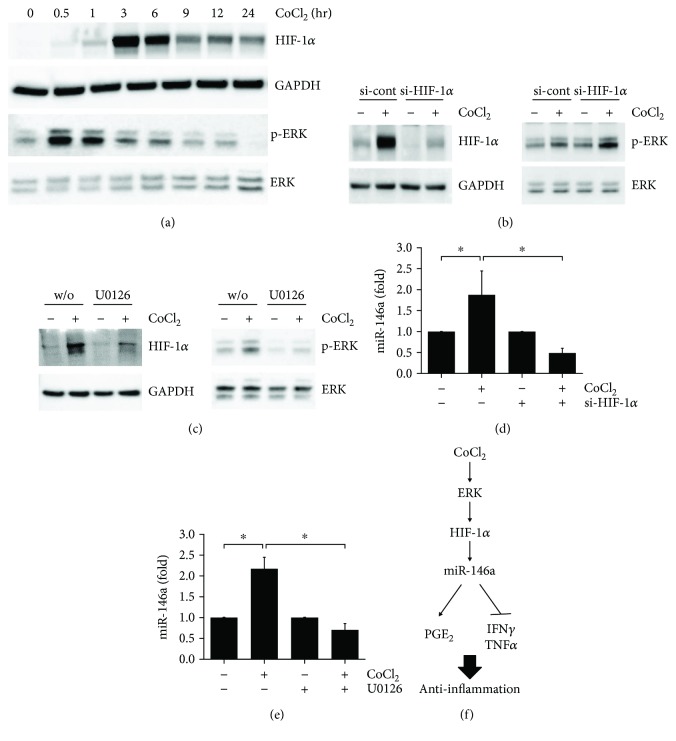
Cobalt chloride (CoCl_2_) stimulates the immunomodulation of hUCB-MSCs through an ERK- and HIF-1*α*-dependent pathway. (a) hMSCs were treated with 100 *μ*M CoCl_2_ and then harvested at the indicated times. The protein level of HIF-1*α* and p-ERK was determined by Western blot analysis. (b) At 48 hr posttransfection with control siRNA (CTRL) or HIF-1*α*-specific siRNA (si-HIF-1*α*), hUCB-MSCs were treated with 100 *μ*M CoCl_2_ for 30 min or 3 hr. (c) hUCB-MSCs were treated with 100 *μ*M CoCl_2_ for 30 min or 3 hr in the absence or presence of 10 *μ*M U0126. ERK phosphorylation and HIF-1*α* expression were determined by Western blot analysis. (d) hUCB-MSCs were transfected with control siRNA (si-control) or HIF-1*α* siRNA (si-HIF-1*α*) and then treated with 100 *μ*M CoCl_2_ for 72 h. (e) hUCB-MSCs were pretreated with the vehicle control (DMSO) or the ERK-specific inhibitor 10 *μ*M U0126 for 60 min and then treated with 100 *μ*M CoCl_2_ for 72 h. The expression level of miR-146a was determined by RT-qPCR. (f) Schematic illustration of the molecular mechanisms involved in the CoCl_2_-enhanced anti-inflammatory effects. Data represent the mean ± SD, *n* = 3; ^∗^ *p* < 0.05. P: hPBMCs; P^∗^: allogeneic hPBMCs as stimulator; H: phytohemagglutinin; MSC: naïve hUCB-MSCs; CoMSC: CoCl_2_-pretreated hUCB-MSCs.

It is well known that CoCl_2_ is able towards transcriptional factor HIF-1 (HIF-1) activation by hypoxia [17]. Moreover, it has been reported that hypoxia condition induces the activation of the ERK pathway, which is involved in HIF-1*α* expression [18]. To explore whether CoCl_2_ induced ERK phosphorylation and HIF-1*α* expression, hUCB-MSCs were treated with 100 *μ*M CoCl_2_ for the indicated time periods. As shown in Figure 4(a), hUCB-MSC increased the expression of HIF-1*α* in a time-dependent manner upon treatment of 100 *μ*M CoCl_2_ and reached the maximum level at 3 hr, and ERK phosphorylation was time-dependent and occurred rather early. Therefore, we next examined the effects of HIF-1*α* knockdown by treatment of hUCB-MSCs with siRNA, followed by culture under CoCl_2_-treated conditions. The silencing of HIF-1*α* significantly inhibited CoCl_2_-induced HIF-1*α* expression (Figure 4(b)), as well as regulation of PGE_2_, TNF-*α*, and IFN-*γ* secretion (Supplementary Figure 3). As shown in Figure 4(c), pretreatment with U0126 prevented CoCl_2_-induced ERK phosphorylation. In addition, to determine whether the ERK pathway also affects HIF-1*α*, pretreatment with U0126 significantly inhibited CoCl_2_-induced HIF-1*α* expression. By contrast, the CoCl_2_-induced ERK phosphorylation was not changed by silencing of HIF-1*α* (Figure 4(b)). Also, CoCl_2_-induced immunomodulation of hUCB-MSCs, such as the regulation of PGE_2_, TNF-*α*, and IFN-*γ* secretion, was blocked by U0126 (Supplementary Figure 4). To elucidate the underlying mechanism, the role of ERK and HIF-1*α* signaling in CoCl_2_-induced miR-146a expression was investigated. The treatment of hUCB-MSCs with CoCl_2_ increased miR-146a expression, which was significantly inhibited by silencing of HIF-1*α* (Figure 4(d)) and U0126 (Figure 4(e)). As summarized in Figure 4(f), these results suggested that miR-146a plays a critical role in CoCl_2_-induced anti-inflammatory effects through the ERK- and HIF-1*α*-dependent signaling pathway.

### 3.5. CoCl_2_ Enhanced the Therapeutic Effects of hUCB-MSCs for the Treatment of Inflammatory Disease

**Figure 5:**
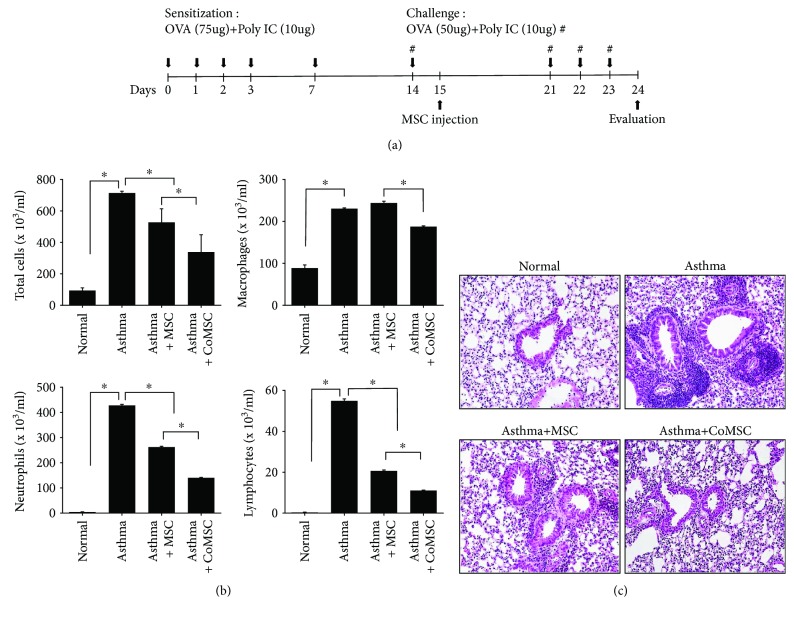
Administration of cobalt chloride- (CoCl_2_-) treated hUCB-MSCs inhibited airway inflammation in a mouse model of asthma. (a) Schematic of the experimental studies. (b) BALF cell differentials in naïve hUCB-MSCs, CoCl_2_-treated hUCB-MSCs, and PBS-treated asthma mouse models. (c) Representative photomicrographs stained with hematoxylin and eosin depicting histologic inflammation on day 24 for asthma models are depicted for each experimental condition. ^∗^ *p* < 0.05. MSC: naïve hUCB-MSCs; CoMSC: CoCl_2_-pretreated hUCB-MSCs.

As intravenously injected MSCs are retained for a short period in the lungs, where they exert anti-inflammatory effects, a well-established mouse model of asthma was used to compare the effects of naïve MSCs and CoCl_2_-treated MSCs. Six-week-old wild-type BalB/C mice were sensitized with the allergen OVA and synthetic dsRNA [poly(I:C)], subsequently challenged with OVA and poly(I:C) for 10 days, and evaluated 24 h after the final challenge, as shown in Figure 5(a). Cellularity in BALF showed that lung infiltration of inflammatory cells, such as macrophages, neutrophils, and lymphocytes, strongly decreased in the CoCl_2_-MSC-injected mice compared with the naïve MSC-injected mice (Figure 5(b) and Supplementary Figure 5). The inhibitory effects of hMSC administration were also evident histologically, as lung tissue sections from CoCl_2_-hMSC-treated mice showed greater reduction in inflammatory cells in the airway tissues compared with naïve MSCs (Figure 5(c)).

## 4. Discussion

Various source-derived MSCs have the ability to modulate the regenerative environment via anti-inflammatory and immunomodulatory mechanisms and are therefore considered to be a good candidate for cell-based therapies. Although adult bone marrow (BM) and adipose tissue (AT) are main source of MSCs for clinical use, they are limited because of the stringent requirements for autologous donors. hUCB-MSCs have a higher rate of cell proliferation, lower senescence, and more extensive anti-inflammatory effects than BM-MSCs and AT-MSCs, along with accessibility, making hUCB-MSCs more suitable than other MSCs for clinical applications [19].

Prostaglandin E_2_ (PGE_2_) plays a key role in association of anti-inflammation and immune suppression via EP_4_ receptor activation [20]. PGE_2_ produced by MSCs exerts anti-inflammatory effects through the regulation of immune cell activation and maturation [21]. The results of the present study demonstrated that pretreatment with CoCl_2_ significantly increased the anti-inflammatory potency of hUCB-MSCs, as evidenced by the increased expression of the anti-inflammatory mediator PGE_2_ and decreased expression of the proinflammatory factors TNF-*α* and INF-*γ*. CoCl_2_ is a hypoxia-mimetic compound that activates HIF-1*α* and other signaling pathways [10, 17]. Furthermore, siRNA-mediated knockdown of HIF-1*α* attenuated the CoCl_2_-induced anti-inflammatory effects.

Mitogen-activated protein kinases (MAPKs) play an important role in numerous cellular processes, which are regulated by various extracellular stimuli, such as cytokines, stress, and growth factors [22]. The results of the present study also showed that CoCl_2_-induced HIF-1*α* expression required activation of ERK. Also, CoCl_2_-induced secretion of anti-inflammatory cytokines was prevented by pretreatment of hUCB-MSCs with the ERK inhibitor U0126, indicating the involvement of ERK in CoCl_2_-induced anti-inflammatory effects.

miRs are small, single-stranded RNA molecules of 21–23 nucleotides in length that fully or partially bind to their target mRNA and posttranscriptionally regulate the expression of target genes by inducing decay of target mRNA or suppressing translation. Recently, miRs have been found to be an important beneficial mechanism in the immune microenvironment. For example, the macrophage inflammatory response to infection involves the upregulation of several miRs, such as miR-155, miR-146, miR-147, miR-21, and miR-9. It has been reported that the induction of miR-29 suppresses host immune response by targeting IFN-*γ*. Furthermore, miRs have a strong impact on the immunomodulatory activity of MSCs [23–25].

Additional studies are warranted to dissect the effect of specific miRs on the regulatory function of MSC. We hypothesized that miRs could control the inflammation processes of hUCB-MSCs. To examine this possibility, the potential of miR to regulate hUCB-MSCs in an inflammatory environment was analyzed using the miR array, which revealed altered expression of more than 49 miRs between populations of hUCB-MSCs and hPBMCs + hUCB-MSCs.

The miR-146 family is composed of two members: miR-146a and miR-146b. The roles of miR-146 in the suppression of inflammatory cytokine secretion and negative regulation of inflammation induced via the innate immune response have been demonstrated [25–28]. Interestingly, treatment with CoCl_2_ induced miR-146a expression in hUCB-MSCs, which suppressed the expression of inflammatory factors. Furthermore, overexpression of pre-miR-146a induced the anti-inflammatory potency of hUCB-MSCs. These findings demonstrated that miR-146a creates an anti-inflammatory environment and is required for the effect of CoCl_2_. Furthermore, MAPKs were shown to positively regulate miR-146a expression, and CoCl_2_-induced activation of ERK had a significant effect on miR-146a induction in hUCB-MSCs. These results indicate that CoCl_2_ treatment induces anti-inflammation through ERK-HIF-1*α*-miR-146a expression in hUCB-MSCs. Although further studies are necessary to elucidate the mechanisms underlying this process, these findings indicate that miR-146a is a novel target of CoCl_2_ and a key regulator of the CoCl_2_-enhanced anti-inflammatory potency of hUCB-MSCs. These findings present clues to further understand the immunomodulation role of miR-146a in hUCB-MSCs, and activators of miR-146a expression may be used to develop anti-inflammatory therapeutics. In addition, pretreatment with CoCl_2_ had no significant effect on the stem cell properties of hUCB-MSCs, such as morphology, growth, stem cell marker expression, and differentiation abilities. CoCl_2_ preconditioning enhanced the anti-inflammatory capacity and improved the therapeutic effects of hUCB-MSCs compared with naïve MSCs in an *in vivo* asthma model. These results suggest that CoCl_2_ signaling may improve the therapeutic effects of hUCB-MSCs, which may be a very useful model for the clinical application of allogeneic cell therapies.

## 5. Conclusions

In conclusion, we demonstrated that the treatment with CoCl_2_ enhanced the anti-inflammatory property of hUCB-MSCs through ERK-HIF-1*α*-dependent miR-146a expression. CoCl_2_-induced high potency MSCs showed therapeutic effects more than naïve MSCs in the asthma models. These findings suggest that pretreatment of hUCB-MSCs with CoCl_2_ improves the therapeutic effects of MSCs for the clinical application of allogeneic cell therapies.

## Figures and Tables

**Table 1:** List of upregulated miRNAs (>2-fold).

Upregulated		
miRNA	Fold change	*p* value
hsa-miR-146a	53.58	0.0003
hsa-miR-29b	9.76	0.007
hsa-miR-139-5p	6.79	0.011
hsa-miR-936	4.61	0.065
hsa-miR-147b	4.54	0.143
hsa-miR-122	4.13	0.012
hsa-miR-1972	3.68	0.053
hsa-miR-29c	3.37	0.036
hsa-let-7i^∗^	3.16	0.005
hsa-miR-155	3.10	0.053
hsa-miR-4748	2.79	0.030
hsa-miR-29a	2.74	0.039
hsa-miR-146b-5p	2.73	0.003
hsa-miR-212	2.72	0.103
hsa-miR-17^∗^	2.46	0.002
hsa-miR-1207-5p	2.42	0.015
hsa-miR-30e	2.41	0.038
hsa-miR-424	2.31	0.139
hsa-miR-1246	2.28	0.361
hsa-miR-4513	2.08	0.015
hsa-miR-424^∗^	2.05	0.279

**Table 2:** List of downregulated miRNAs (>2-fold).

Downregulated		
miRNA	Fold change	*p* value
hsa-miR-149	6.06	0.011
hsa-miR-654-3p	3.52	0.003
hsa-miR-758	3.41	0.018
hsa-miR-130b^∗^	3.40	0.022
hsa-let-7e^∗^	3.37	0.028
hsa-let-7a-2^∗^	3.30	0.004
hsa-miR-355	3.21	0.027
hsa-miR-411^∗^	3.14	0.016
hsa-miR-379^∗^	3.00	0.047
hsa-miR-708	3.00	0.721
hsa-miR-27b	2.77	0.015
hsa-miR-23c	2.70	0.004
hsa-miR-369-5p	2.60	0.030
hsa-miR-302d	2.52	0.468
hsa-miR-422a	2.45	0.074
hsa-miR-4485	2.45	0.065
hsa-miR-137	2.41	0.019
hsa-miR-412	2.38	0.049
hsa-miR-2355-3p	2.26	0.004
hsa-miR-409-3p	2.22	0.033
hsa-miR-496	2.20	0.049
hsa-miR-23b^∗^	2.19	0.022
hsa-miR-584	2.15	0.003
hsa-miR-217	2.12	0.089
hsa-miR-140-5p	2.09	0.058
hsa-miR-23b	2.07	0.000
hsa-miR-4324	2.05	0.026
hsa-miR-744^∗^	2.01	0.004

## Supplementary Materials

Supplementary Materials Figure 1: dose and time dependency of CoCl_2_ treatment in the immunogenicity of hUCB-MSCs using the MLR assay. Supplementary Figure 2: flow cytometry analysis of naïve human umbilical cord blood-derived hUCB-MSCs and CoCl_2_-treated hUCB-MSCs. Supplementary Figure 3: the role of HIF-1*α* in CoCl_2_-induced immunomodulation of hUCB-MSCs such as PGE_2_, TNF-*α*, and IFN-*γ* regulation. Supplementary Figure 4: the role of ERK in CoCl_2_-induced immunomodulation of hUCB-MSCs such as PGE_2_, TNF-*α*, and IFN-*γ* regulation. Supplementary Figure 5: BALF cell differentials in naïve hUCB-MSCs, CoCl_2_-treated hUCB-MSCs, and PBS-treated asthma mouse models. Data are presented as the percent of the total cell count.
