# Supplementary figures and images for: Cobalt Chloride Enhances the Anti-Inflammatory Potency of Human Umbilical Cord Blood-Derived Mesenchymal Stem Cells through the ERK-HIF-1α-MicroRNA-146a-Mediated Signaling Pathway
Source: Stem Cells Int. 2018 Sep 5;2018:4978763. doi: 10.1155/2018/4978763 (PMC6145052; doi:10.1155/2018/4978763)

Supp. Fig 1

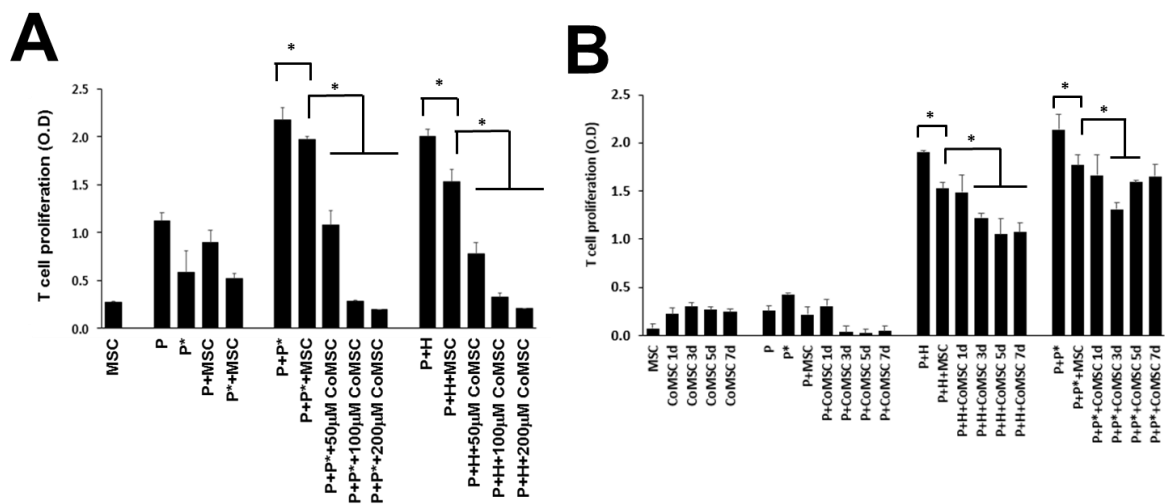

Supp. Fig 2

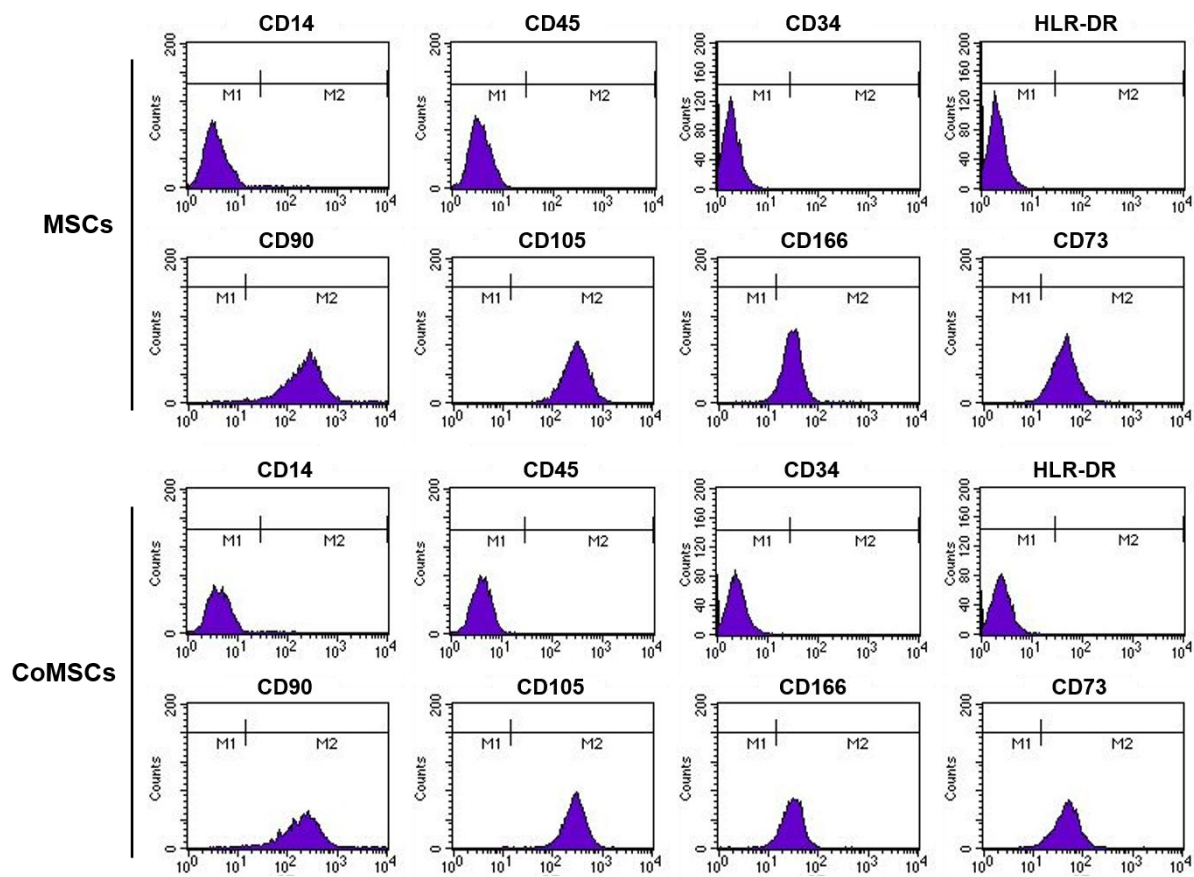

Supp. Fig 3

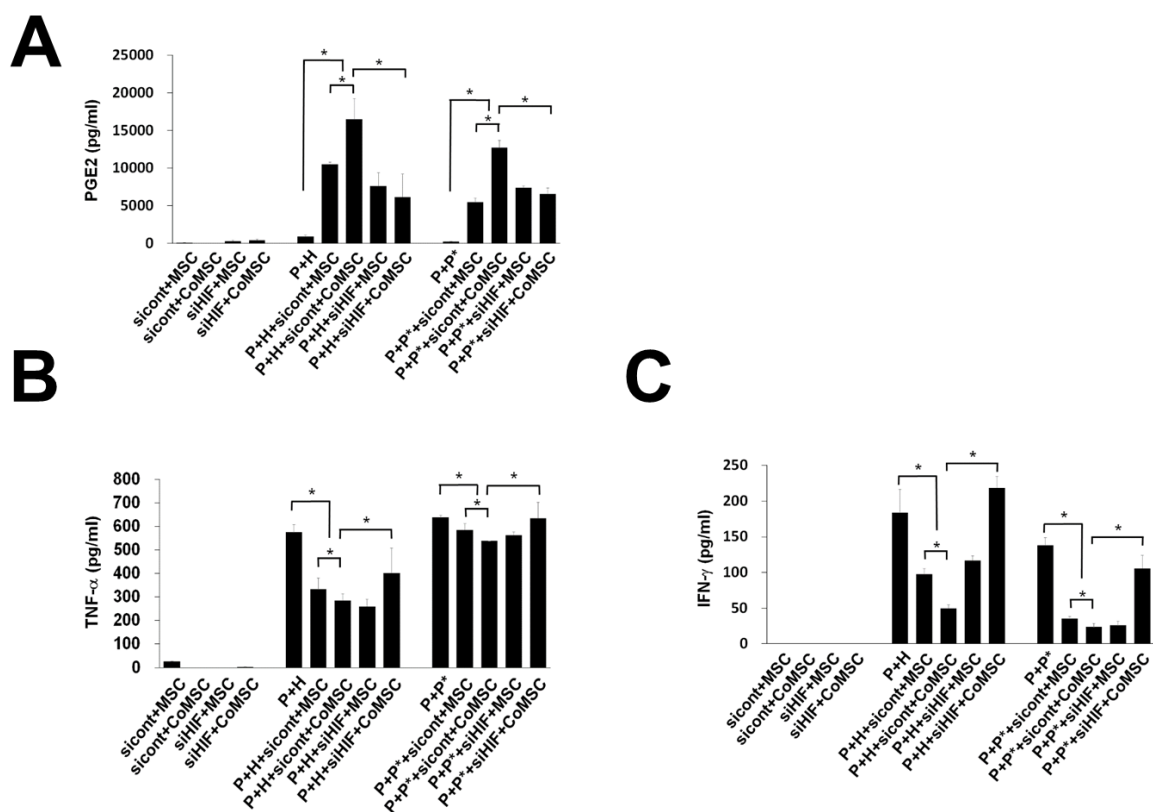

Supp. Fig 4

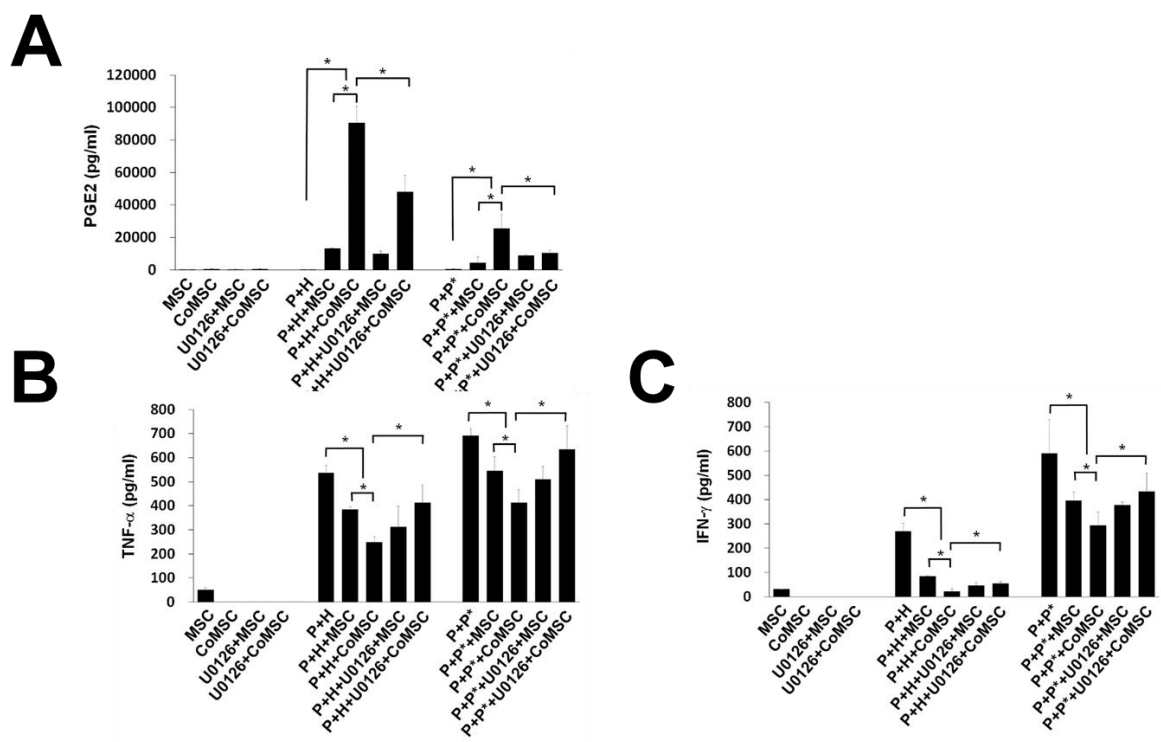

Supp. Fig 5

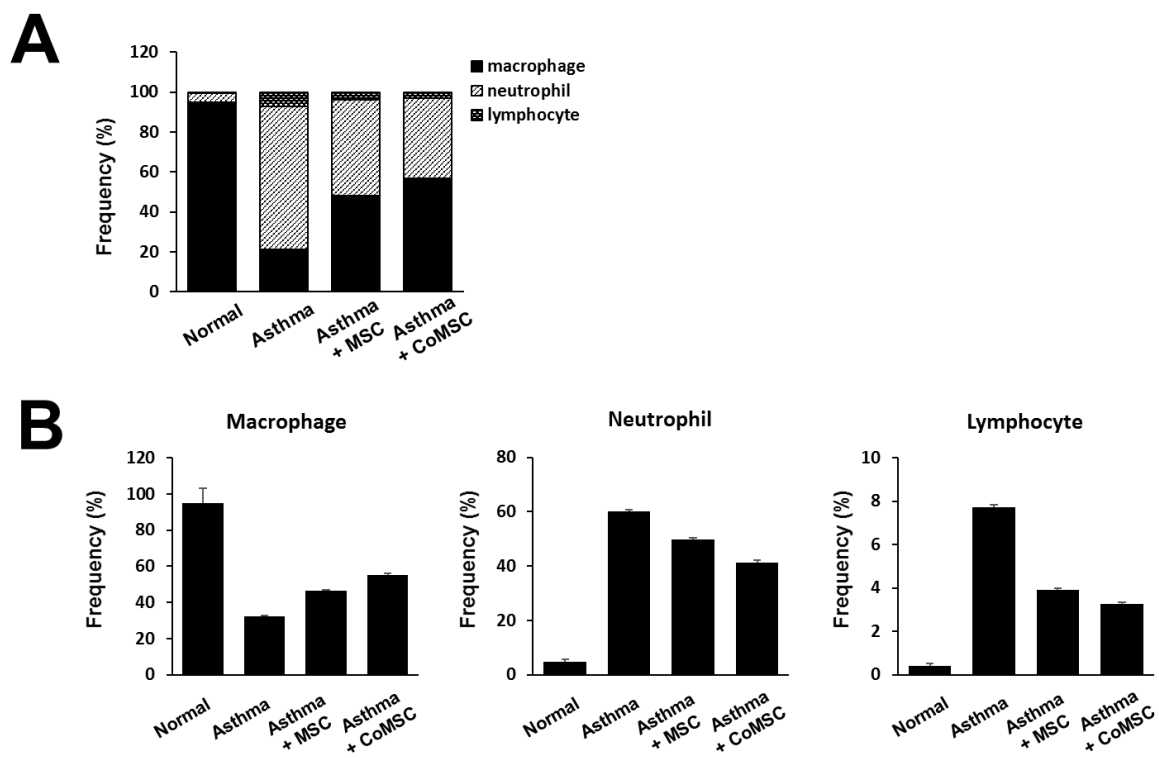

Supplement: Supplementary Materials — Figure 1: dose and time dependency of CoCl2 treatment in the immunogenicity of hUCB-MSCs using the MLR assay. Supplementary Figure 2: flow cytometry analysis of naïve human umbilical cord blood-derived hUCB-MSCs and CoCl2-treated hUCB-MSCs. Supplementary Figure 3: the role of HIF-1α in CoCl2-induced immunomodulation of hUCB-MSCs such as PGE2, TNF-α, and IFN-γ regulation. Supplementary Figure 4: the role of ERK in CoCl2-induced immunomodulation of hUCB-MSCs such as PGE2, TNF-α, and IFN-γ regulation. Supplementary Figure 5: BALF cell differentials in naïve hUCB-MSCs, CoCl2-treated hUCB-MSCs, and PBS-treated asthma mouse models. Data are presented as the percent of the total cell count. [file 4978763.f1.pdf]
